# Feasibility of using *P16* methylation as a cytologic marker for esophageal squamous cell carcinoma screening: A pilot study

**DOI:** 10.1002/cam4.4718

**Published:** 2022-03-29

**Authors:** Zhiyuan Fan, Yu Qin, Jing Zhou, Ru Chen, Jianhua Gu, Minjuan Li, Jiachen Zhou, Xinqing Li, Dongmei Lin, Jinwu Wang, Dajun Deng, Wenqiang Wei

**Affiliations:** ^1^ Office of National Central Cancer Registry, National Cancer Center/National Clinical Research Center for Cancer/Cancer Hospital Chinese Academy of Medical Sciences and Peking Union Medical College Beijing China; ^2^ Key Laboratory of Carcinogenesis and Translational Research (MOE/Beijing), Division of Etiology Peking University Cancer Hospital and Institute Beijing China; ^3^ Department of Epidemiology and Biostatistics, School of Public Health Xi'an Jiaotong University Health Science Center Xi'an China; ^4^ Department of Pathology Peking University Cancer Hospital Beijing China; ^5^ Department of Pathology Linzhou Cancer Hospital Linzhou China

**Keywords:** cytology, early detection, esophageal squamous cell carcinoma, minimally invasiveness, *P16* methylation

## Abstract

**Background:**

Early diagnosis and treatment of esophageal squamous cell dysplasia (ESCdys) and esophageal squamous cell carcinoma (ESCC) could significantly reduce the incidence and mortality of ESCC. This pilot study aimed to investigate whether *P16/CDKN2A* methylation could serve as a cytologic biomarker for early detection of ESCdys and ESCC.

**Methods:**

Paired esophageal biopsy and cytology specimens (exfoliated cells) were obtained from subjects at different stages of ESCC development. The methylation status of *P16* gene in these two specimen types was determined using a 115‐bp MethyLight assay. Categorical data were compared by the Chi‐square test. Logistic regression was performed to assess adjusted odds ratios of *P16* methylation associated with ESCC and ESCdys. Prediction models for identifying individuals at risk of ESCC and high‐grade ESCdys (high‐grade intraepithelial neoplasia, HGIN) were developed by multivariable logistic regression. Diagnostic performance was evaluated using receiver operating characteristic (ROC) analysis. Internal validation of the prediction models was performed using the 1000‐bootstrap resample.

**Results:**

A total of 105 subjects with diagnoses ranging from normal mucosa through ESCC were included in this study. An increase in *P16* methylation frequency was observed with increasing severity of esophageal lesions (*p* for trend <0.001). In the adjusted logistic regression models, *P16* methylation in cytology specimens was positively associated with ESCC and ESCdys risk, whereas *P16* methylation in biopsy specimens was only associated with a higher risk of developing ESCC. The predictive capacity of base model I (AUC, 0.816) for ESCC and HGIN was significantly increased by adding *P16* methylation in cytology specimens (model III; AUC, 0.882; *p* = 0.043), but not *P16* methylation in biopsy specimens (model II; AUC, 0.850; *p* = 0.225). Bootstrap validation showed optimism‐corrected AUC of 0.789 for model I, 0.822 for model II, and 0.854 for model III.

**Conclusion:**

*P16* methylation as a cytologic marker was associated with the ESCC development and has the potential for application in minimally invasive ESCC screening.

## Graphical abstract

*P16* methylation as a cytologic marker has the potential for application in minimally invasive screening for esophageal squamous cell carcinoma.

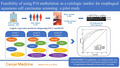

## 1 INTRODUCTION

Esophageal cancer is one of the most aggressive tumors worldwide, ranking the seventh most common cancer and the sixth leading cause of cancer death.^1^ Approximately 54% of global cases occur in China with a dominant type of esophageal squamous cell carcinoma (ESCC).^2^, ^3^ Patients with precursor lesions and early ESCC are always asymptomatic, therefore most ESCC patients are diagnosed at advanced stages, resulting in a 5‐year survival rate of 30.3%.^4^ However, patients detected at an earlier stage have a 5‐year survival rate of up to 85%.^5^ Therefore, early detection and appropriate treatment of curable precursors and early‐stage ESCC are of utmost importance for reducing ESCC morbidity and improving overall survival.^6^

Esophageal squamous cell dysplasia (ESCdys), including low‐ and high‐grade intraepithelial neoplasia (LGIN and HGIN), are the precursor lesions associated with the risk of developing ESCC, and increasing grades of ESCdys are associated with increased risk of ESCC.^7^, ^8^ A multicenter population‐based cohort study involving 637,500 high‐risk subjects demonstrated that endoscopic screening can effectively reduce the cumulative incidence and mortality of ESCC by 26% and 57%, respectively.^9^ However, the compliance rate of endoscopic examination remains low due to its high cost and invasiveness, which limit its application as a widespread screening tool for ESCC.^10^ This emphasizes the need for an effective and minimally invasive test to facilitate early ESCC screening. Several non‐endoscopic sampling methods have been developed and tested in the high‐incidence areas of China, including esophageal balloon cytology and liquid‐based balloon cytology.^11^, ^12^, ^13^ Exfoliated cells from esophagus mucosa were collected by these non‐endoscopic devices and applied to cytological diagnosis of ESCC and ESCdys. Unfortunately, despite the significant advantages of minimally invasiveness and cost‐effectiveness, these methods were inadequate for population‐based screening due to their low sensitivity.^14^

Recent advances have demonstrated that combining non‐endoscopic sampling devices with DNA methylation markers enables an efficient and accurate method of screening at‐risk populations for Barrett's esophagus (BE), which has sparked interest in their application in ESCC screening.^15^, ^16^ Aberrant methylation of *P16*/*CDKN2A* in biopsy specimens was widely considered to be correlated with the development and pathogenesis of ESCC, and may have great potential for early ESCC detection.^17^, ^18^ However, it is unknown whether *P16* methylation as a cytologic marker could be applied to population‐based ESCC screening. Therefore, we conducted this pilot study to evaluate the feasibility of using *P16* methylation as a cytologic biomarker for early detection of ESCC and ESCdys, and to provide new insight into ESCC screening strategies.

## 2 METHODS

### 2.1 Study subjects and specimen collection

This study was carried out in Linzhou Cancer Hospital, Henan province, a high‐risk area of ESCC in China. The target population consisted of ESCC outpatients and residents who underwent endoscopic screening from April 2019 to June 2019.

Subjects were excluded if they had:

1. a contraindication for endoscopy;
2. prior history of chemotherapy or radiotherapy to the chest;
3. underwent endoscopic treatment for esophageal cancer.

Demographic characteristics and exposure information were collected for each subject using a uniform questionnaire, including age, sex, education level, income, smoking, alcohol and tea consumption, family history of cancer, and dietary habits.

All subjects underwent endoscopic examination according to the National Esophageal Cancer Screening guideline.^19^ Lugol's iodine solution (1.2%) was used to stain the entire esophagus mucosa, leaving dysplastic lesions unstained. Cytology specimens were obtained from unstained suspicious lesions using endoscopy‐directed brushings (to roughly simulate non‐endoscopic sampling device) and then stored in PreservCyt solution (Cytyc Corporation, Marlborough, MA) at −80°C until analysis (Figure S1). Then biopsy specimens were taken from the same lesions immediately. Biopsy specimens were fixed in 10% buffered formalin, embedded in paraffin, and stained with hematoxylin and eosin (HE). Biopsy slides were reviewed independently by two well‐trained pathologists (DL and JW) to ensure the histological diagnosis. This study was approved by Institutional Review Board of the Cancer Hospital of the Chinese Academy of Medical Sciences (No.15–151/1078). Written informed consent was obtained from all subjects before specimen collection.

### 2.2 DNA preparation

#### 2.2.1 Specimen preparation

The formalin‐fixed and paraffin‐embedded biopsies were sectioned at 5 μm, and eight to 10 biopsy sections were deparaffinized in xylene (twice for 10 min) and rehydrated with graded ethanol (100% and 80%).

The frozen cytology specimens were thawed at room temperature. After well mixed, the solution (200 μl) was centrifuged (12,000 rpm, 10 min) and the supernatant was decanted.

#### 2.2.2 DNA extraction

Genomic DNA of esophageal biopsies or exfoliated cells was extracted manually using a DNA extraction kit according to the manufacturer's protocol (Com Win Biotech, China). The extracted DNA was eluted in 50 μl Tris & EDTA (TE) buffer eventually. DNA concentration was measured by spectrophotometer and all DNA samples had OD260/280 value between 1.7 and 1.9. 1% agarose gel electrophoresis was used to assess DNA integrity and no DNA degradation was detected.

### 2.3 Bisulfite modification

Genomic DNA was modified with sodium bisulfite using the EZ DNA Methylation‐Gold Kit (Zymo Research) following the manufacturer's instructions. Briefly, a total of 20 μl of genomic DNA was mixed with 130 μl of CT Conversion Reagent, and then denatured at 98°C for 10 min, followed by incubation at 64°C for 2.5 h, and 4°C for 30 min. Next, M‐biding buffer (600 μl) was added into the Zymo spin column and centrifuged for 30 s, followed by addition of 100 μl M‐Wash buffer into the column and centrifugation was performed for another 30 s. After 200 μl of M‐sulphonation buffer and 200 μl of M‐wash buffer were carried out, 30 μl of M‐elution buffer was used to elute the DNA sample.

### 2.4 Quantification of *P16* methylation using the MethyLight assay

We used a useful and practical assay, 115‐bp MethyLight assay, to detect *P16* methylation for clinical diagnosis.^20^ CpG island within *P16* exon‐1 is the sequence in which *P16* methylation could be stably maintained.^21^ The 115‐bp methylated amplicon in *P16* exon‐1 was analyzed to quantify the proportion of methylated *P16* alleles by the assay. Briefly, a forward primer (5′‐cgcggtcgtggttagttagt‐3′), a reverse primer (5′‐tacgctcgacgactacgaaa‐3′), and a methylated‐*P16*‐specific probe (5’‐6FAM‐gttgtttttcgtcgtcggtt‐TAMRA‐3′) were used to detect copy number of the 115‐bp methylated *P16* templates. *COL2A1* was selected as the reference gene with a forward primer (5′‐tctaacaattataaactccaaccaccaa‐3′), a reverse primer (5′‐gggaagatgggatagaagggaatat‐3′), and a *COL2A1*‐specific probe (5’‐6FAM‐ccttcattctaacccaatacctatcccacctctaaa‐BHQ‐1‐3′). An ABI7500 thermal cycler was used to conduct the PCR reactions with 45 cycles, and the fluorescence value was detected at 58.5°C. PCR products (5 μl) were also checked in polyacrylamide gel electrophoresis (PAGE) analysis.

### 2.5 Quality control of qPCR panel

Three replicate samples were carried out in parallel, and samples were considered positive if the cycle threshold (Ct) value of 2 or 3 replications were less than 40. To avoid the false‐negative detection, the results of *P16* methylation analyses were considered valid when Ct value of *COL2A1* is less than 29.3.^22^ Genomic DNA samples from colon cancer cell lines RKO and gastric cancer cell lines MGC803 were used as methylated‐*P16* positive and negative controls, respectively.

### 2.6 Statistical analysis

All statistical analyses were performed using Statistic Package for Social Science (SPSS) version 23.0, GraphPad Prism 8.0, and R software version 4.0.5. Categorical variables were compared by the Chi‐square or Fisher's exact test. Odds ratios (ORs) with 95% confidence intervals (95% *CI*s) of *P16* methylation associated with ESCC and ESCdys were calculated by logistic regression models, which were adjusted by four well‐established risk factors of ESCC, that is, age, sex, smoking and alcohol consumption, taking the normal and esophagitis subjects as the reference group.^23^ Z‐tests were performed to compare these ORs at each lesion grade to test the difference in the *P16* methylation between these two specimens. Then, three prediction models were developed for estimating ESCC and HGIN risk by multivariable logistic regression. Eleven conventional variables based on literature review were selected as candidate predictors, including age, sex, education level, annual income per capita, cigarette smoking, alcohol and tea consumption, family history of cancer, and dietary habits of consuming fresh fruits, pickled, or hot food. The base model I was constructed based on the 11 variables with a forward stepwise selection method using *p*‐values of 0.05 and 0.10 as cutoffs for the entry and departure, respectively. The variables that remained significant in the base model I were then included as covariates, and *P16* methylation in biopsy and cytology specimens were added to the model to develop model II and III, respectively. The goodness‐of‐fit was evaluated by Hosmer–Lemeshow test. Diagnostic performance was evaluated by the receiver operating characteristic (ROC) curve. The area under the ROC curve (AUC) was reported and compared by the DeLong method. Finally, internal validation of the three prediction models was performed using the 1000‐bootstrap resample. All statistical tests were two‐sided, and the results were statistically significant if *p* < 0.05.

## 3 RESULTS

### 3.1 Study participants

A total of 105 subjects were included in this pilot study, including 83 cases recruited based on endoscopy screening project and 22 ESCC from outpatient. Among these 83 cases, 32 individuals were diagnosed without pathological changes, 30 esophagitis, 12 LGIN, eight HGIN, and one ESCC. Due to insufficient ESCC samples, 22 tumor samples were obtained from outpatients to cover the research needs. All ESCC patients enrolled in the study were diagnosed with early‐stage to mid‐stage cancers. HE stained sections and endoscopic images were represented in Figure S2.

To investigate the association of baseline characteristics with the development of ESCC and HGIN, a univariate analysis was conducted. As shown in Table 1, the ESCC and HGIN patients were more often male, older, smokers, consumed alcohol, tea and pickled food more frequently, and have a family history of cancer. In addition, subjects with a lower education level, income and intake of fresh fruit appear to be at a higher risk of developing ESCC and HGIN. We also investigated whether there was an association between baseline characteristics and *P16* methylation. Age was found significantly correlated with *P16* methylation both in the two specimen types. Intake of fresh fruit and pickled food were not significantly associated with the *P16* methylation in biopsy specimens, however, the two variables were significantly associated with *P16* methylation in cytology specimens (Table S1).

**TABLE 1:** Baseline characteristics of study participants

Variables	Normal mucosa (*n* = 32)	Esophagitis (*n* = 30)	LGIN (*n* = 12)	HGIN (*n* = 8)	ESCC (*n* = 23)	*p*‐value
Age (years)						0.026
<60	20	16	3	4	5	
≥60	12	14	9	4	18	
Sex						0.008
Female	18	23	6	6	5	
Male	14	7	6	2	18	
Education						0.009
Primary education or less	13	17	7	4	20	
Secondary education or more	19	13	5	4	3	
Annual income per capita						0.004
≤10,000 RMB	15	18	7	6	20	
>10,000 RMB	17	12	5	2	3	
Drinking alcohol						0.004
Yes	4	3	0	0	10	
No	28	27	12	8	13	
Smoking						<0.001
Yes	6	3	1	0	15	
No	26	27	11	8	8	
Drinking tea						0.047
Yes	5	0	0	0	7	
No	27	30	12	8	16	
Family history of cancer						0.029
Yes	18	19	9	7	19	
No	14	11	3	1	4	
Taking fruit						0.021
High	14	10	5	2	3	
Low	18	20	7	6	20	
Taking pickled food						0.050
High	5	8	4	2	11	
Low	27	22	8	6	12	
Taking hot food						0.335
High	19	23	11	6	19	
Low	13	7	1	2	4	

*Note*: *p*‐values were calculated by the Chi‐square test, comparing the normal, esophagitis & LGIN group and HGIN & ESCC group.

Abbreviations: ESCC, esophageal squamous cell carcinoma; HGIN, high‐grade intraepithelial neoplasia; LGIN, low‐grade intraepithelial neoplasia.

### 3.2 *P16* methylation

**FIGURE 1:**
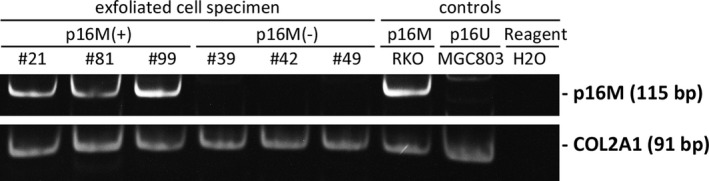
The polyacrylamide gel electrophoresis (PAGE) image of the Methylight analysis to detect the methylation status of *P16* gene in six representative samples. Genomic DNA samples of RKO and MGC803 cells were used as the methylated‐P16 positive and negative controls (p16M and p16U). *COL2A1* was used as the reference gene

According to lesion characteristics, specimens were categorized into four main pathology categories: no dysplasia (including normal esophagus and esophagitis), LGIN, HGIN, and ESCC. The results for representative specimens in the MethyLight analysis are presented in Figure 1. *P16* methylation frequency in biopsy specimens showed an increasing trend with the increasing severity of histological diagnosis (*p* for trend <0.001). *P16* methylation was detected in 1.6% biopsies from subjects with no dysplasia, 8.3% with LGIN, 12.5% with HGIN, and 30.4% with ESCC (*p* < 0.001 compared to no dysplasia). For the diagnosis of ESCC and HGIN, *P16* methylation in biopsy specimens had a sensitivity of 25.8% and an AUC of 0.616 (95% CI, 0.489–0.742).

Then methylation status of *P16* CpG island in the paired cytology specimens was further measured. Once again, we noticed an increasing trend in *P16* methylation frequency in cytology specimens with increasing severity of esophageal lesions (*p* for trend <0.001). *P16* methylation was observed in 4.8% subjects with no dysplasia and 25.0% subjects with LGIN. Those patients with HGIN and ESCC had a significantly higher frequency of *P16* methylation (37.5% and 43.5%, respectively; *p* < 0.050 compared to no dysplasia). When targeted at ESCC and HGIN, the diagnostic sensitivity of *P16* methylation in cytology specimens was 41.9% and the AUC was 0.669 (95% CI, 0.546–0.792). Although *P16* methylation occurred more frequently in cytology specimens than that in biopsy specimens, the difference at each stage of ESCC development was not statistically significant (Table 2).

**TABLE 2:** Frequency of *P16* methylation in biopsy and cytology specimens

Diagnosis	*n*	Biopsy specimens		Cytology specimens		*p*‐value (biopsy vs. Cytology)
		Case with *P16* methylation (%)	*p*‐value	Case with *P16* methylation (%)	*p‐*value	
No dysplasia	62	1 (1.6)		3 (4.8)		0.611^b^
LGIN	12	1 (8.3)	0.300^a^	3 (25.0)	0.050^a^	0.590^b^
HGIN	8	1 (12.5)	0.217^a^	3 (37.5)	**0.017** ^a^	0.569^b^
ESCC	23	7 (30.4)	**<0.001** ^a^	10 (43.5)	**<0.001** ^a^	0.359^b^
Trend		*p* for trend **<0.001**		*p* for trend **<0.001**		

No dysplasia, normal esophagus and esophagitis; LGIN, low‐grade intraepithelial neoplasia; HGIN, high‐grade intraepithelial neoplasia; ESCC, esophageal squamous cell carcinoma.

The bold values indicate *p* < 0.05.

^a^ Compared with no dysplasia.

^b^ Compared between biopsy specimen and cytology specimen.

**FIGURE 2:**
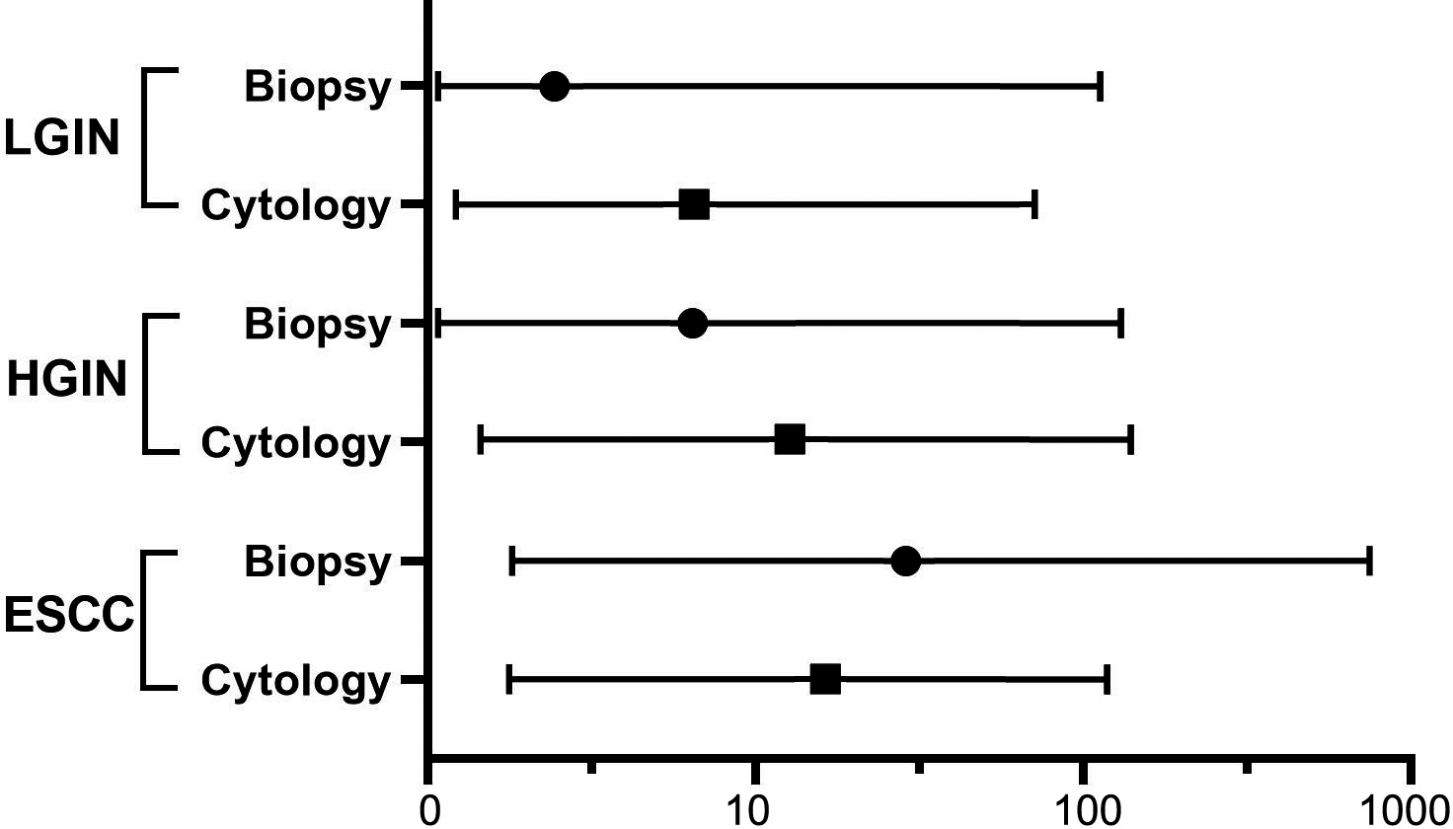
The association between *P16* methylation and the risk for different grades of esophageal dysplasia. The odds ratio (OR) and 95% confidence intervals (*CI*s) were obtained from logistic regression analysis adjusted for age, sex, smoking, and alcohol consumption. Black circles represent ORs for *P16* methylation in biopsy specimens. Black boxes represent ORs for *P16* methylation in cytology specimens. Horizontal whiskers represent 95% *CI*s. LGIN, low‐grade intraepithelial neoplasia; HGIN, high‐grade intraepithelial neoplasia; ESCC, esophageal squamous cell carcinoma

In the adjusted logistic regression models, *P16* methylation in biopsy specimens was significantly associated with the risk of ESCC (adjusted OR = 48.9; 95% CI, 3.2–738.1; *p* = 0.005) but not of LGIN or HGIN (*p* > 0.050). However, *P16* methylation in cytology specimens was associated not only with higher ESCC risk (adjusted OR = 20.6; 95% *CI*, 3.1–137.2; *p* = 0.002), but also with increased risk of LGIN (adjusted OR = 8.6; 95% *CI*, 1.0–73.1; *p* = 0.048) and HGIN (adjusted OR = 18.5; 95% CI, 2.1–160.1; *p* = 0.008), implying better representativeness of cytology specimens than biopsy specimens for detection of *P16* methylation in ESCC development. However, the differences in *P16* methylation between two kinds of specimens at each lesion grade were not statistically significant, and the confidence intervals became relatively wide due to the small number of cases for this sub‐analysis (Figure 2).

### 3.3 Diagnostic performance of risk prediction models

**FIGURE 3:**
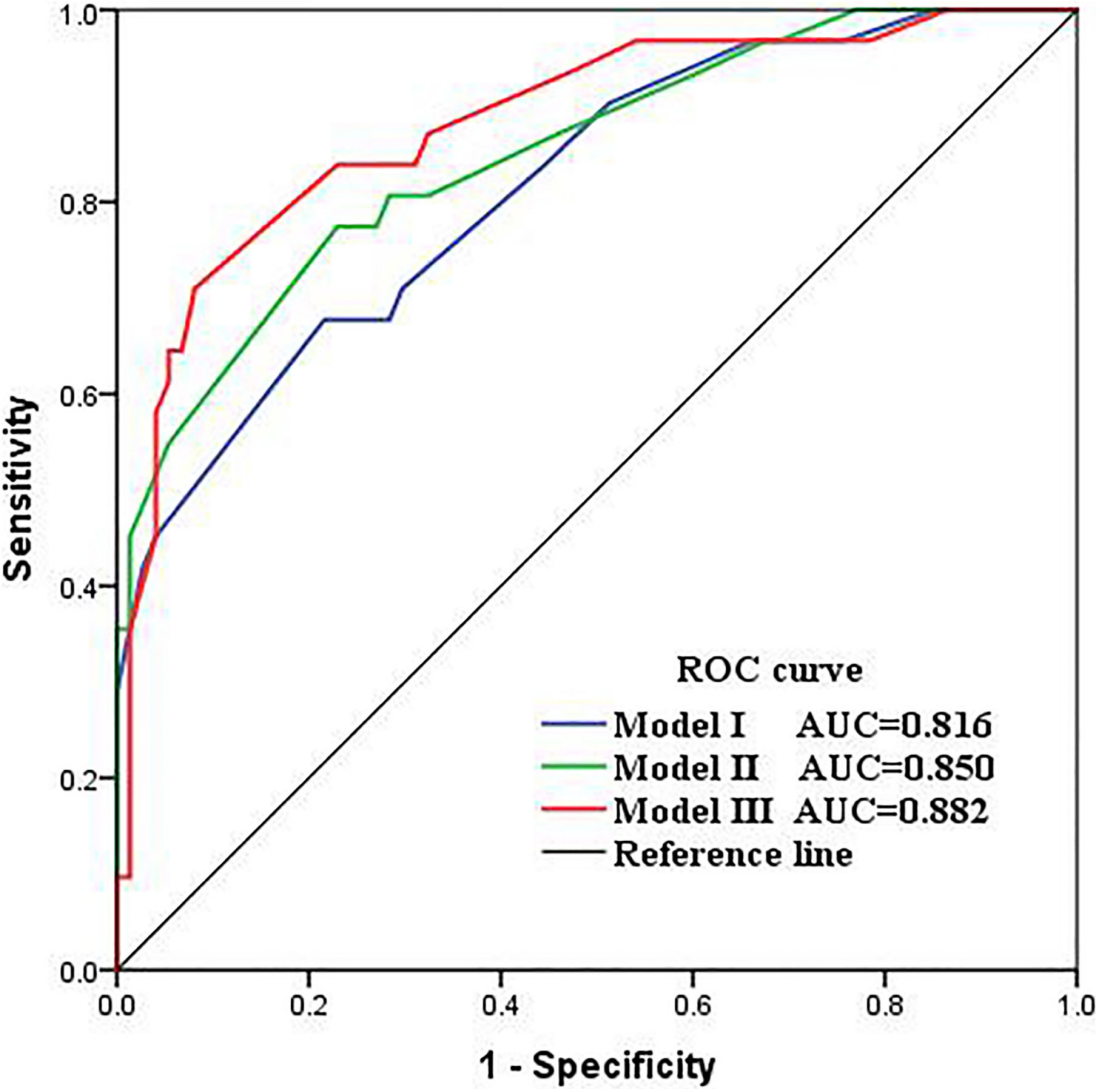
Receiver operating characteristic (ROC) curves of the prediction models for diagnosing ESCC and HGIN. Model I: base model; Model II: four variables included in the base model + *p16* methylation in biopsy specimens; Model III: four variables included in the base model + *p16* methylation in cytology specimens. AUC, area under the ROC curve; ESCC, esophageal squamous cell carcinoma; HGIN, high‐grade intraepithelial neoplasia

As shown in Table 3, the base prediction model included four variables: education level, annual income per capita, smoking, and family history of cancer. Higher education level and income were correlated with decreased risk of ESCC and HGIN, whereas cigarette smoking and family history of cancer were associated with increased risk of ESCC and HGIN. In model II and III, *P16* methylation was found to be a significant independent predictor for developing ESCC and HGIN. In addition, the Hosmer–Lemeshow goodness‐of‐fit tests had *p*‐value >0.20, indicating that all three models fitted the data well. Table 4 reports the diagnostic performance of the multivariate logistic regression models. The base model had relatively fair predictive capacity (AUC, 0.816) for ESCC and HGIN, which was significantly increased by adding *P16* methylation in cytology specimens (model III; AUC, 0.882; *p* = 0.043). The diagnostic performance of model II (AUC, 0.850), which incorporated *P16* methylation in biopsy specimens, was better than the base model I and worse than model III, but these did not reach statistical significance (*p* = 0.225 and *p* = 0.298, respectively) (Figure 3). Internal validation by bootstrapping analysis showed optimism‐corrected AUC of 0.789 for the model I, 0.822 for the model II, and 0.854 for the model III.

**TABLE 3:** Variables associated with ESCC and HGIN risk in the multivariable logistic models

Variables	Adjusted OR (95% CI)		
	Model I	Model II	Model III
Education			
Primary education or less	1.00 (reference)	1.00 (reference)	1.00 (reference)
Secondary education or more	0.26 (0.09–0.80)	0.21 (0.06–0.70)	0.31 (0.10–1.02)
Annual income per capita			
≤10,000 RMB	1.00 (reference)	1.00 (reference)	1.00 (reference)
>10,000 RMB	0.21 (0.06–0.70)	0.21 (0.06–0.73)	0.19 (0.05–0.70)
Smoking			
No	1.00 (reference)	1.00 (reference)	1.00 (reference)
Yes	8.46 (2.56–27.98)	8.93 (2.35–33.97)	9.41 (2.57–34.40)
Family history of cancer			
No	1.00 (reference)	1.00 (reference)	1.00 (reference)
Yes	4.49 (1.23–16.37)	4.93 (1.18–20.60)	3.65 (0.92–14.49)
*P16* methylation in biopsy specimens			
Negative	—	1.00 (reference)	—
Positive	—	15.85 (2.21–113.68)	—
*P16* methylation in cytology specimens			
Negative	—	—	1.00 (reference)
Positive	—	—	8.95 (2.25–35.56)

Abbreviations: 95% CI, 95% confidence interval; ESCC, esophageal squamous cell carcinoma; HGIN, high‐grade intraepithelial neoplasia; OR, odds ratio.

**TABLE 4:** Diagnostic performance of prediction models

Performance index	Models for ESCC & HGIN		
	Model I	Model II	Model III
Sensitivity	67.7%	77.4%	71.0%
Specificity	78.4%	77.0%	91.9%
AUC (95% *CI*)	0.816 (0.727–0.905)	0.850 (0.768–0.933)	0.882 (0.807–0.956)

*Notes*: Model I: base model; Model II: four variables included in the base model + *P16* methylation in biopsy specimens; Model III: four variables included in the base model + *P16* methylation in cytology specimens.

Abbreviations: 95% CI, 95% confidence interval; AUC, area under the receiver operating characteristic curve; ESCC, esophageal squamous cell carcinoma; HGIN, high‐grade intraepithelial neoplasia.

## 4 DISCUSSION

ESCC is a fatal disease with a poor prognosis due to the lack of symptoms at early stages.^4^ Population‐wide endoscopic screening for ESCC is not realistic due to many limitations including invasiveness, high cost, and lack of expert clinicians in developing regions.^10^ Non‐endoscopic sampling devices in combination with accurate biomarkers show promise for overcoming these limitations. In this pilot study, we found an increasing trend for the *P16* methylation frequency with disease development in both biopsy and cytology specimens. We noticed that *P16* methylation in cytology specimens was a significant predictor of ESCC and its precursor lesions, but *P16* methylation in biopsy specimens was only associated with ESCC risk, although the differences between the two specimens did not reach statistical significance. Furthermore, the predictive capacity of base model for ESCC and HGIN was significantly increased by adding *P16* methylation in cytology specimens, but not *P16* methylation in biopsy specimens. These preliminary findings suggest that *P16* methylation may be a potential biomarker for non‐endoscopic ESCC screening.

*P16* is a cyclin‐dependent kinase inhibitor that induces cell cycle arrest at the G1 phase by preventing the phosphorylation of retinoblastoma protein (RB1), thereby negatively regulating the cell cycle progression.^24^ *P16* methylation has been recognized as a potential biomarker for early detection of many cancer types and found to be associated with malignant transformation of epithelial dysplasia in gastric and oral mucosa in several prospective studies.^25^, ^26^ In ESCC, it was found that *P16* methylation occurred in early lesions and increased more significantly in higher‐grade lesions.^18^

Our results reinforced the notion that *P16* methylation was a frequent and early event in ESCC development.^27^ We observed a significant increase in the *P16* methylation frequency from no dysplasia to ESCC, which is at the same level as other findings.^28^, ^29^ Notably, we found that *P16* methylation in cytology specimens was more sensitive than that in biopsy specimens for diagnosing ESCC and ESCdys, although the differences at each stage of ESCC development indicated no significant difference. There are two possible explanations for these observations. First, this is a pilot study with a relatively small sample size and further studies with larger sample sizes are required to validate our current findings. Second, the inconsistence between the two specimen types may due to the impact of intratumoral heterogeneity on *P16* methylation in biopsy specimens. Previous studies showed a wide variant of *P16* methylation frequency in ESCC biopsy ranging from 19% to 88%, which may be explained by potential sampling bias caused by spatial intratumoral heterogeneity and the under‐representation of one single tumor biopsy to assessment.^30^, ^31^, ^32^ Interestingly, the exfoliated cells sampling device could overcome the limitation by covering the entire lesion area to decrease sampling bias.^33^ These findings suggested that the representativeness of cytologic specimens may be better than that of biopsies for *P16* methylation detection in ESCC and precursor lesions.

Non‐endoscopic sampling devices coupled with cytological diagnosis have been studied extensively as minimally invasive methods for ESCC screening since 1990s.^11^, ^12^ Due to poor accuracy, however, none of these researches achieved the target for improving a screening program at the population level. Although the role of *P16* methylation in ESCC development has been studied intensively, only one previous study examined *P16* methylation in cytology specimens and reported that *P16* methylation was observed in 9% subjects with no dysplasia, 8% with LGIN, 14% with HGIN.^34^ In contrast, our study showed a considerably higher sensitivity for identifying ESCdys and an increasing trend for *P16* methylation frequency with pathological progression, which is consistent with other studies using biopsy specimen.^29^, ^35^ Differences in sampling devices or methylation detection technology may account for the better results in our study.

Recently, newer non‐endoscopic cytological sampling devices have been developed for the diagnosis of BE and esophageal adenocarcinoma.^36^ A multicenter randomized controlled trial confirmed the feasibility, tolerability, and safety of the novel device.^37^ Several studies have indicated that endoscopic brushings could simulate non‐endoscopic cytological screening devices and the combination of non‐endoscopic sampling devices with DNA methylation markers provides a highly accurate and cost‐effective screening procedure that could be clinically useful for BE screening.^38^, ^39^ This innovative procedure has opened up the prospect of improved availability and cost‐effectiveness of screening for ESCdys and early‐stage ESCC. This study used endoscopy‐directed brushings to roughly simulate non‐endoscopic sampling devices to collect cytology specimens, and found that *P16* methylation in cytology specimens might have greater potential clinical utility in ESCC screening than that in biopsy specimens. It should be noted that *P16* methylation alone was inadequate for ESCC screening, but these results provided preliminary evidence for the feasibility of methylated genes as cytologic markers for ESCC screening. Further investigation of methylated marker panels is warranted.

The present study has several strengths. To our knowledge, this is the first study to report that *P16* methylation in cytology specimens may have better diagnostic performance than that in biopsy specimens for ESCC and its precursor lesions. Paired biopsy and cytology specimens were collected from the same subjects which could largely minimize the individual difference between samples. Finally, the MethyLight assay that we developed allows a more accurate detection for the methylation status of *P16* CpG island around transcription start site.

Our study was designed as a pilot study with encouraging results but limitations. First, the sample size was relatively small and it was from one hospital, which limits the extrapolation of our results. In addition, the number of events per variable was small in the logistic regression models which may result in bias in regression coefficients. Second, dysplastic or ESCC cells collected by endoscopy‐directed brushings might be much more concentrated than that by non‐endoscopic sampling devices. Thus, results from different devices are not fully comparable. Third, we only examined the status of *P16* methylation and it would be necessary to explore multiple biomarkers to compensate for the sensitivity and specificity limitations of a single biomarker. This study is a preliminary evaluation of the feasibility of *P16* methylation as a cytologic biomarker for primary ESCC screening. In the future, we will identify sensitive DNA methylation marker panels and combine with non‐endoscopic cytological screening devices to validate the present results in a large prospective study.

In summary, we demonstrated the feasibility of *P16* methylation as a cytologic marker for early detection of ESCC and its precursor lesions. Our findings provided preliminary evidence that combining DNA methylation markers and non‐endoscopic cytological screening devices may be a promising approach for large‐scale ESCC screening of at‐risk population. Future large prospective multicenter studies are required to validate this approach.

## CONFLICT OF INTEREST

The authors declare no potential conflict of interest.

## AUTHOR CONTRIBUTIONS

WW, DD, and YQ designed the study. ZF, YQ, and JZ carried out the experiments. DL and JW reviewed the pathological slides. ZF, YQ, and JG performed the statistical analysis and discussed the results. ZF, YQ, JZ, ML, and XL contributed to questionnaire information and specimen collection. ZF wrote the manuscript with support from YQ, WW, and DD, and all authors read and approved the final manuscript.

## ETHICAL STATEMENT

The study was conducted according to the guidelines of the Declaration of Helsinki and approved by the Institutional Review Board of the Cancer Hospital of the Chinese Academy of Medical Sciences (No.15–151/1078). Written informed consent was obtained from all participants.

## Supporting information


Data S1

## Data Availability

Source data are available from the corresponding author on reasonable request.
