# Supplementary material for: Feasibility of using P16 methylation as a cytologic marker for esophageal squamous cell carcinoma screening: A pilot study
Source: Cancer Med. 2022 Mar 29;11(21):4033–42. doi: 10.1002/cam4.4718 (PMC9636512; doi:10.1002/cam4.4718)
Supplement: Supplementary file 1 — Data S1 [file CAM4-11-4033-s001.docx]

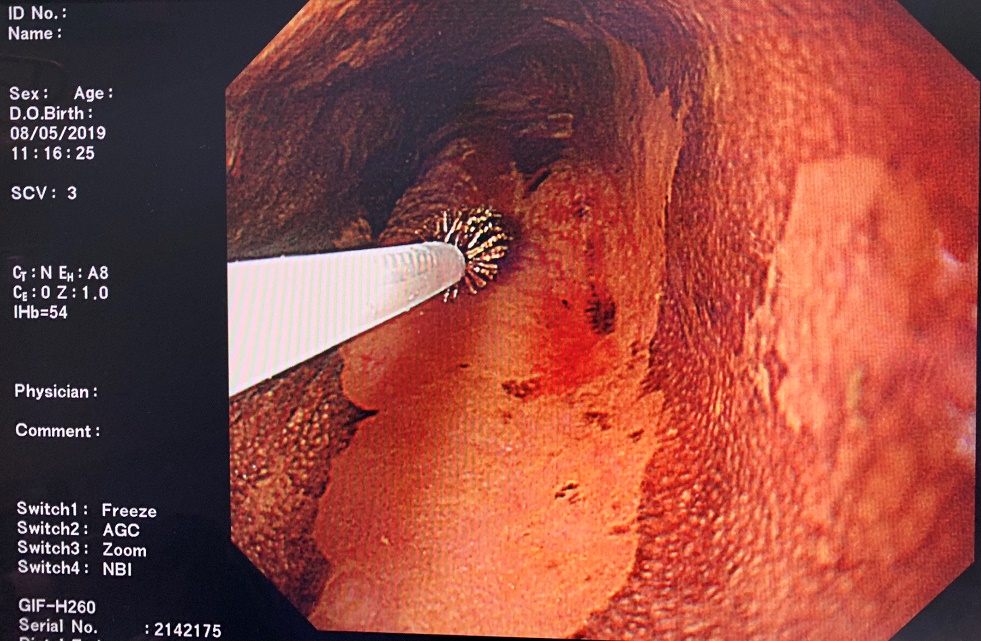


**Supplementary Figure S1.** Administration and passage of the endoscopy-directed brushings to obtain a sample of esophageal epithelial cells.


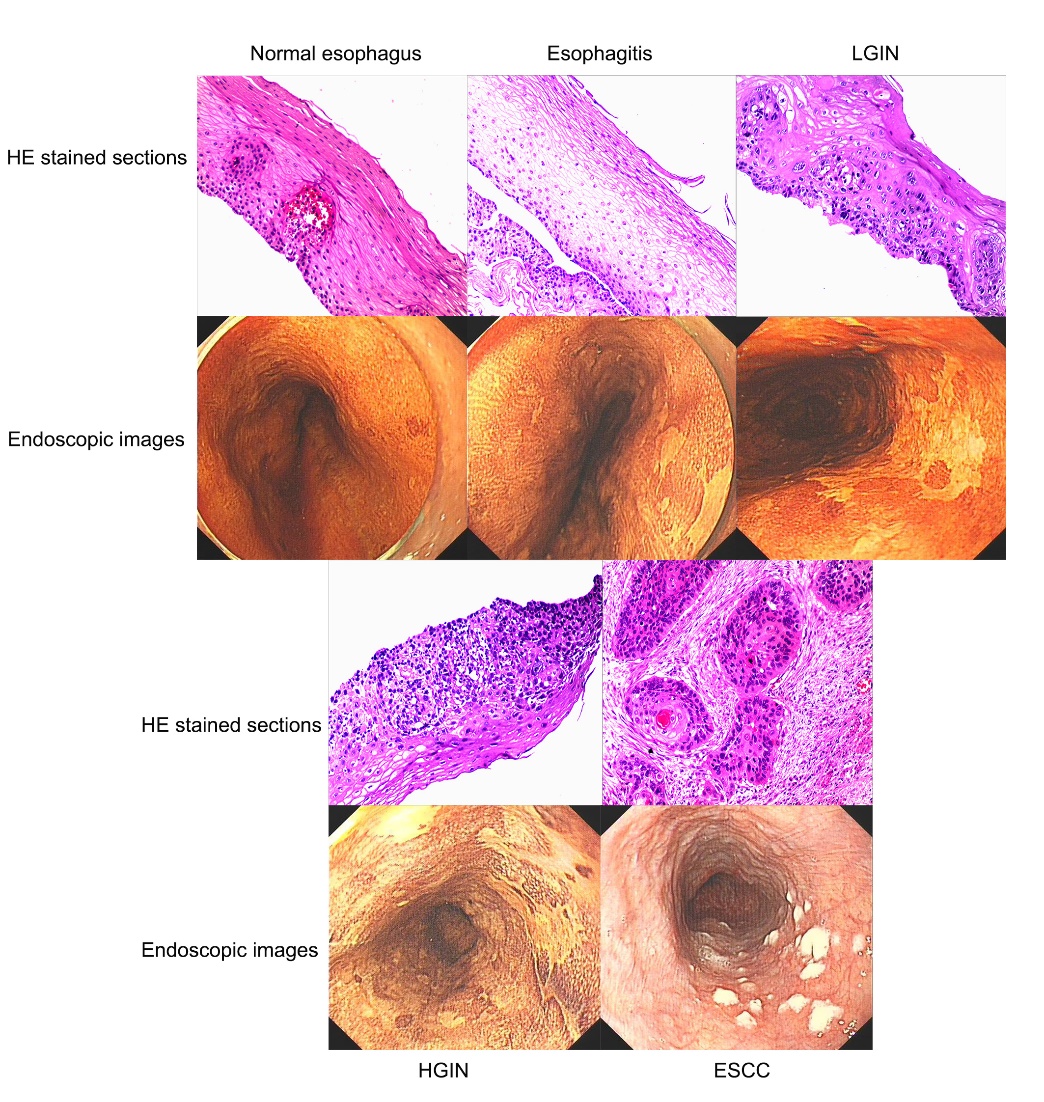
**Supplementary Figure S2.** Hematoxylin-eosin (H&E) stained sections (100×) and endoscopic images from five representative subjects with normal esophagus, esophagitis, LGIN, HGIN, and ESCC. LGIN, low-grade intraepithelial neoplasia; HGIN, high-grade intraepithelial neoplasia; ESCC, esophageal squamous cell carcinoma.

**Supplementary Table 1.** Association of *P16* methylation with baseline characteristics

| **Variables** | **Category** | ***n*** | **Biopsy specimens** | | **Cytology specimens** | | |
| --- | --- | --- | --- | --- | --- | --- | --- |
|  |  |  | **Case with *P16* methylation (%)** | ***p-*value** | | **Case with *P16* methylation (%)** | ***p-*value** |
| **Age (years)** | <60 | 48 | 1 (2.1) | **0.040** | | 3 (6.3) | **0.004** |
|  | ≥60 | 57 | 9 (15.8) |  | | 16 (28.1) |  |
| **Sex** | Female | 58 | 5 (8.6) | 0.987 | | 11 (19.0) | 0.797 |
|  | Male | 47 | 5 (10.6) |  | | 8 (17.0) |  |
| **Education** | Primary education or less | 61 | 6 (9.8) | 1.000 | | 14 (23.0) | 0.128 |
|  | Secondary education or more | 44 | 4 (9.1) |  | | 5 (11.4) |  |
| **Annual income per capita** | ≤10,000 RMB | 66 | 8 (12.1) | 0.403 | | 13 (19.7) | 0.579 |
|  | >10,000 RMB | 39 | 2 (5.1) |  | | 6 (15.4) |  |
| **Drinking alcohol** | Yes | 17 | 3 (17.6) | 0.427 | | 5 (29.4) | 0.327 |
|  | No | 88 | 7 (8.0) |  | | 14 (15.9) |  |
| **Smoking** | Yes | 25 | 5 (20.0) | 0.098 | | 6 (24.0) | 0.561 |
|  | No | 80 | 5 (6.3) |  | | 13 (16.3) |  |
| **Drinking tea** | Yes | 12 | 2 (16.7) | 0.709 | | 4 (33.3) | 0.290 |
|  | No | 93 | 8 (8.6) |  | | 15 (16.1) |  |
| **Family history of cancer** | Yes | 72 | 8 (11.1) | 0.645 | | 16 (22.2) | 0.105 |
|  | No | 33 | 2 (6.1) |  | | 3 (9.1) |  |
| **Taking fruit** | High | 34 | 2(5.9) | 0.600 | | 1(2.9) | **0.005** |
|  | Low | 71 | 8(11.3) |  | | 18(25.4) |  |
| **Taking pickled food** | High | 30 | 5(16.7) | 0.227 | | 11(36.7) | **0.002** |
|  | Low | 75 | 5(6.7) |  | | 8(10.7) |  |
| **Taking hot food** | High | 78 | 8(10.3) | 0.957 | | 16(20.5) | 0.422 |
|  | Low | 27 | 2(7.4) |  | | 3(11.1) |  |
